# Beyond the Debye length in high ionic strength solution: direct protein detection with field-effect transistors (FETs) in human serum

**DOI:** 10.1038/s41598-017-05426-6

**Published:** 2017-07-12

**Authors:** Chia-Ho Chu, Indu Sarangadharan, Abiral Regmi, Yen-Wen Chen, Chen-Pin Hsu, Wen-Hsin Chang, Geng-Yen Lee, Jen-Inn Chyi, Chih-Chen Chen, Shu-Chu Shiesh, Gwo-Bin Lee, Yu-Lin Wang

**Affiliations:** 1 Institute of Nanoengineering and Microsystems, National Tsing Hua University, Hsinchu, 300 Taiwan, R.O.C.; 2 Department of Power Mechanical Engineering, National Tsing Hua University, Hsinchu, 300 Taiwan, R.O.C.; 3 Institute of Biomedical Engineering, National Tsing Hua University, Hsinchu, 300 Taiwan, R.O.C.; 4 Department of Electrical engineering, National Central University, Jhongli City, Taoyuan County 320 Taiwan, R.O.C.; 5 Department of Medical Laboratory Science and Biotechnology, National Cheng Kung University, Tainan City, 701 Taiwan, R.O.C.

## Abstract

In this study, a new type of field-effect transistor (FET)-based biosensor is demonstrated to be able to overcome the problem of severe charge-screening effect caused by high ionic strength in solution and detect proteins in physiological environment. Antibody or aptamer-immobilized AlGaN/GaN high electron mobility transistors (HEMTs) are used to directly detect proteins, including HIV-1 RT, CEA, NT-proBNP and CRP, in 1X PBS (with 1%BSA) or human sera. The samples do not need any dilution or washing process to reduce the ionic strength. The sensor shows high sensitivity and the detection takes only 5 minutes. The designs of the sensor, the methodology of the measurement, and the working mechanism of the sensor are discussed and investigated. A theoretical model is proposed based on the finding of the experiments. This sensor is promising for point-of-care, home healthcare, and mobile diagnostic device.

## Introduction

Field-effect transistors (FETs) attract great interest for biomolecular detection, due to their high sensitivity, small size, and label-free detection, which are suitable for point-of-care or personal homecare devices. Either planar or nanowire FET-based biosensors have been widely studied using various materials, such as Si^1^, GaN^2^, carbon nanotube (CNT)^3^, or graphene oxide^4^. Conventionally, FET-based biosensors with receptors (ex. antibody) immobilized on the gate region above the active channel of the FETs face an intrinsic issue, which is the severe charge screening effect in high ionic strength solutions, such as in serum or blood samples, leading to low sensitivity for direct detection of protein in the physiological environment. The Debye length in physiological salt environment (1X PBS) is near 0.7 nm, which is much smaller than the size of a regular IgG antibody (5~10 nm)^5^. In order to effectively detect proteins with receptor-immobilized FETs, the electrical measurements are usually conducted in diluted buffer solutions, such as in 0.1X PBS or 0.01X PBS, where the Debye lengths as 2.4 nm and 7.4 nm, respectively^1, 6, 7^. However, diluted ionic strength solution may cause the change in protein structure, resulting in the loss of protein activity, and the binding affinity as well. For most biological reactions, which occur in physiological high salt environment, a biosensor that can be used directly with physiological samples is much favored. Besides, an additional washing process is needed for conventional FET-based biosensors to remove the unbound antigen before electrical measurement, which also increases the complexity of the whole sensor system. Therefore, direct detection of the target protein in physiological sample is very demanding.

Previously, several groups have reported that conventional FET-based biosensors can effectively detect proteins in physiological salt environment, using alternative current (AC) signals in drain-source voltage (V_ds_), in conjunction with a reference electrode, in a relatively high frequency^8–11^. The better sensitivity of AC signals compared to that of DC signals, was explained with the breakdown of the electric-double-layer (EDL) near the surface of the FET channel, due to fast switching of the direction of the applied bias, leading to deeper penetration of the electric potential of the target protein, exceeding the regular Debye length^9^. However, the optimized operational frequencies from several groups are quite different, ranging from 1 KHz~50 MHz, based on the results from different groups^8–11^. Besides, AC signals have not been demonstrated in direct protein detection in physiological samples, such as in serum or blood. The role of the reference electrode in the AC bias for conventional FET-based sensors is ambiguous, because the distance between the reference electrode and the FET channel, the geometry and the surface area of the reference electrode, and the voltage applied on the reference electrode have not been systematically investigated. In fact, when the reference electrode is biased with a voltage, all the above factors should be considered. The detailed mechanism and how the ions or biomolecules react with the AC bias are still mysterious.

In this study, we propose a new type of FET-based biosensor, with a new design of the sensor and a new methodology of electrical measurement, and demonstrate that our FET biosensors can directly detect proteins in physiological high ionic strength solutions, including 1X PBS containing 1% BSA and human serum. Our FET biosensors are designed as EDL FETs, where the gate electrode is separated from the active channel of the FET and immobilized with antibody or aptamer. The effects of the gap between the gate electrode and the active channel, the open area on the gate electrode, the gate and the drain-source voltages, and different ionic strength solutions are all investigated. In our sensor measurement, the drain current is measured in time domain with only one short pulse bias, in 50 µs with a sampling rate of 10 ns. The measured current was then integrated with time for 50 µs. EDL FETs have been used to improve the performance of FETs, due to the extremely high charge density caused in high ionic strength solution, which induces large change in carrier concentration in the FET channel than the typical dielectric such as SiO_2_^12–16^. EDL FETs have also been reported for pH sensors^17^. However, they have not been reported for biosensors, nor have similar ones shown as ours.

Here we use AlGaN/GaN high electron mobility transistors (HEMTs) for our EDL FET biosensors. AlGaN/GaN HEMT-based biosensors have several advantages. They are chemically inert and thermally stable^18^. Ions in solution may diffuse into SiO_2_, leading to an internal field, which causes the FET performance drift, but ions can hardly diffuse into GaN in solution^19^. The great stability of GaN in harsh environment makes it become a good candidate for biosensors. The advantages and the features of our EDL AlGaN/GaN HEMTs include, (1) direct protein detection in physiological salt environment, such as in 1X PBS and human serum, (2) no dilution and no washing process, which will not affect protein activity and will be suitable for studying biological reactions in real-time, (3) no reference electrode, which simplifies the sensor fabrication and the system, (4) capability to detect charged or un-charged proteins, due to the mechanism of our methodology, surpassing conventional FET biosensor which is believed to detect proteins based on the net charge of proteins (5) fast detection in 5 minutes only, (6) stable baseline, due to the short single pulse, which generates less heat and thermal noise, leading to a steady baseline, (7) excellent repeatability, due to the definition of the signal with the current gain and the total charge from the integration of current with time, instead of the absolute drain current, (8) the adjustable magnitude of the signals by the applied bias, including V_ds_ and V_g_, (9) excellent sensitivity, due to the large amplification of the EDL FET. These advantages and features show that our sensors perform much better than conventional FET-based biosensors.

In this paper, we demonstrate that our EDL AlGaN/GaN HEMTs successfully detect Human Immunodeficiency Virus-1 Reverse Transcriptase (HIV-1 RT), Carcinoembryonic Antigen (CEA), N-terminal pro b-type natriuretic peptide (NT-proBNP), and C-reactive protein (CRP) in 1X PBS containing 1% BSA, and NT-proBNP and CRP in human serum as well, in 5 minutes, with high sensitivity and without any dilution or washing process. The mechanism of our sensors are also investigated and elucidated, with the proposed model. This sensor is promising for the point-of-care, home healthcare, and mobile healthcare devices.

## Results

### Characteristics of Electric-double-layer (EDL) field-effect transistors (FETs)

**Figure 1:**
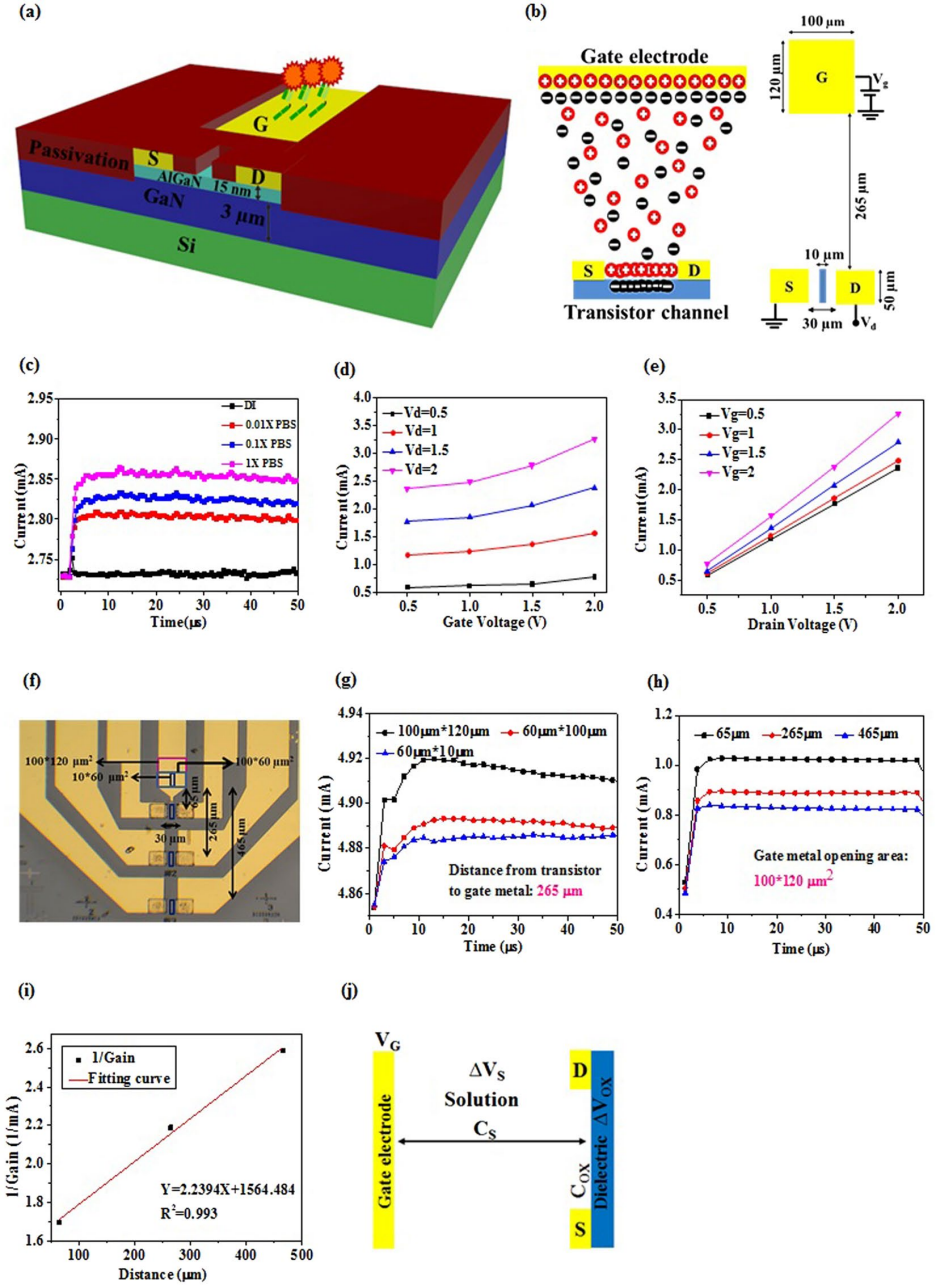
EDL AlGaN/GaN HEMT characteristics. (**a**) Schematic model of functionalized AlGaN/GaN HEMT. The in-plane gate electrode separated from the active channel is functionalized with the receptor. Device is passivated such that only the active channel and functionalized gate electrode are exposed to contact the sample solution. (**b**) Distribution of ions under positive gate bias. Negative ions accumulate on gate electrode surface and simultaneously positive ions accumulate on the active channel surface, increasing the output drain current. EDL formed on both the gate electrode surface and active channel constitute the gate dielectric of FET. Changes in sample solution capacitance thus directly modulate the drain current. The typical gate opening area is 100 µm × 120 µm, distanced at 265 µm from the active channel. (**c**) Different sample solutions containing low to high ion concentrations are tested: De-ionized water, 0.01X PBS, 0.1X PBS and 1X PBS (physiological salt concentration); higher ionic strength leads to increased charge accumulation in the active channel leading to larger drain current. (**d**) The typical HEMT structure is biased at different drain voltages and (**e**) gate voltages. The figures (**d**) and (**e**) show the linear operation of FET, in which transconductance gain is proportional to the applied Vd. (**f**) Top view of EDL HEMT depicting the different geometries designed. (**g**) Comparison of different gate electrode opening areas. When distanced at the same gap from active channel, the larger gate opening area leads to increased drain current. (**h**) Comparison of different gap geometries between gate opening and active channel. With increasing gap distance measured at same bias conditions lead to decreasing drain current. (**i**) The reverse proportionality of increasing gap distance and output current gain is measured and plotted. (**j**)Voltage drop across EDL FET is schematically represented. The applied gate bias drops across the solution and the HEMT dielectric, leading to the formation of EDL constituted solution capacitance. Changes in solution capacitance modulate the output drain current.

EDL AlGaN/GaN HEMTs are designed as a separated gate electrode from the active channel of the HEMTs. The gate electrode and the active channel between source and drain metals are all on the same plane. The HEMT process starts from the mesa formation by ICP etching, followed by source and drain metal deposition with evaporator and thermal annealing to form ohmic contacts. Metal electrodes are then deposited for gate, source and drain electrodes. Finally a passivation layer is covered on the whole device, with lithographic opening on gate electrode and the active channel. Therefore, only the gate electrode and the active channel are exposed in solution dropped on top of the HEMT (Fig. 1(a)). When the gate electrode was applied with a positive bias, negative ions will accumulate on the surface of the gate electrode, in the opened area. At the same time, positive ions will also accumulate on the opening of the active channel, resulting in the increased 2-dimensional electron concentration in the active channel of the HEMT, thus increasing its conductivity (Fig. 1(b)). EDL forms on the gate metal and the surface of the channel as well. Thus, the solution on the top of the FET can be regarded as a part of the gate dielectric of the FET. If the capacitance of the solution changes, the voltage drops in solution and in the solid dielectric of the FET will both change, resulting in different drain current change. Prior to using EDL FETs for biosensors, we first investigate the principle and property of the EDL AlGaN/GaN HEMTs in ionic solutions.

The EDL AlGaN/GaN HEMTs with a typical dimension that has a gap between the gate electrode opening and the active channel as 265 µm, and a gate opening area as 100 µm × 120 µm (Fig. 1(b)), were first measured in different ionic strength solutions, including de-ionized (DI) water, 0.01X PBS, 0.1X PBS, and 1X PBS in time domain, at V_ds_ = 2 V and V_gs_ = 0.5 V, for 50 µs (Fig. 1(c)). The HEMT was biased at V_ds_ = 2 V and V_gs_ = 0 V in the beginning for 2 µs, and then the V_g_ was changed to 0.5 V and maintained for 50 µs at V_ds_ = 2 V, resulting in increased drain current. The increased drain current then quickly relaxes and gets steady. The solution with highest ionic strength causes a highest steady drain current, after V_g_ applied. The DI water caused little current change when V_g_ is applied (Fig. 1(c)). The current change can be regarded as the “gain”, which is an important index (transconductance, g_m_ = dI_d_/dV_g_) indicating the amplification capability of FET. In this paper, we simply use “current gain” to represent the current change after V_g_ is applied. This is definitely different from the “current gain” for bipolar transistors, but is a convenient way to discuss our sensor only. This result indicates that the current gain of the EDL FET is not dominated by the dielectric constant of water, instead, it is determined by the ionic strength, which is attributed to the charges of ions in water, and the ion concentration in solution. When a positive gate voltage is applied, higher ionic strength causes higher density of the negative ion accumulated on the gate electrode, Qn, leading to a higher density of positive ions accumulated on the surface of the active channel of the FET, Qp, resulting in larger increase in the electron concentration, thus causing larger drain current increase (Fig. 1(b)).

The EDL AlGaN/GaN HEMTs were measured in different bias conditions, including V_d_ = 0.5 V~2 V and V_g_ = 0.5 V~2 V, (Fig. 1(d) and (e)) in 1X PBS, with a typical dimension of the HEMT that has a gap between the gate electrode opening and the active channel as 265 µm, and a gate opening area as 120 µm × 100 µm (Fig. 1(b)). The results show that in such a bias condition, the FET is working in the linear region, and the g_m_ is proportional to V_d_, which satisfy the typical FET current-voltage model as shown as in the following equations 1 and 2,

1 $${I}_{d}\cong \frac{W}{L}\mu {C}_{ox}({V}_{g}-{V}_{t}-\frac{1}{2}{V}_{d}){V}_{d}$$

2 $${g}_{m}\equiv {(\frac{\partial {I}_{d}}{\partial {V}_{g}})}_{{V}_{d}}\cong \frac{W}{L}\mu {C}_{ox}{V}_{d}$$

where the *I*_*d*_ is drain current, *W* is the width of the channel, *L* is the length of the gate, *μ* is the electron mobility, *C*_*ox*_ is the dielectric capacitance, *V*_*g*_ is the gate voltage, *V*_*t*_ is the threshold voltage, *V*_*d*_ is the drain voltage, and *g*_*m*_ is the transconductance.

Different geometry of the EDL FETs are designed, fabricated and compared (Fig. 1(f)). Three different gate electrode opening (length × width = L × W), including L × W = 120 µm × 100 µm, 60 µm × 100 µm, and 60 µm × 10 µm with the same gap, 265 µm, between the gate electrode opening and the active channel of the AlGaN/GaN HEMT, are measured and compared at V_d_ = 2 V and V_g_ = 0.5 V (Fig. 1(g)). The HEMT with a larger gate electrode opening has a higher current gain. However, the current gain is not always proportional to the opening area. For example, with the same active channel, the device (L × W = 120 µm × 100 µm) with twice length of another device (L × W = 60 µm × 100 µm), has nearly twice current gain. However, if a device (L × W = 60 µm × 100 µm) has a width 10 times larger than another one (L × W = 60 µm × 10 µm), the current gain only increases 20% (Fig. 1(g)). On the other hand, in another device, if the gate electrode opening is fixed at L × W = 120 µm × 100 µm and the gaps between the gate electrode opening and the active channel are designed as 65 µm, 265 µm and 465 µm, respectively, the current gains measured at V_d_ = 2 V and V_g_ = 0.5 V for these three gaps show reversely proportional relationship (Fig. 1(h) and (i)).

Based on the above results, we can conclude that our devices are ion-gated EDL FETs. The larger gate electrode opening represent that more ions are attracted towards the gate opening, leading to more ions accumulating on the surface of the active channel, which causes increased electron concentration in the channel and increased current gain as well. Previously we have shown that higher salt concentration causes larger current gain (Fig. 1(c)). This can be explained as follows. When a positive V_g_ is applied to the gate electrode, negative ions will be driven by the electric field and tend to move to the gate electrode. Once the steady state is reached, negative ions accumulate on the gate electrodes and no more negative ions from solution will move to the gate electrode. At this moment, near the boundary of the EDL, the drift current of the negative ions, as shown in equation 3, is moving towards the electrode and balanced to the diffusion current, as shown in equation 4, which tends to move away from the gate electrode. The diffusion current is caused by the concentration gradient of negative ions in EDL. We can assume that the negative ion concentration near the EDL boundary is close to the concentration in bulk region. Therefore, the higher salt concentration solution has a higher drift current, which indicates that the diffusion current, or the concentration gradient, is also larger for the high ionic strength solution than that of the lower ionic strength one. The larger concentration gradient represents higher concentration of negative ions on the gate electrode for higher ionic strength solution, leading to higher current gain. Thus, the higher current gain for higher salt concentration solution can be explained.

3 $${J}_{drf}=nZq\mu E$$

4 $${J}_{diff}=-D\frac{dn}{dx}$$

where *J*_*drf*_ and *J*_*diff*_ are the drift and diffusion current density. *n*, *Z*, and *μ* are the ion concentration, valence, and mobility of the negative ion, respectively. *E* is the electric field in solution. D is diffusion coefficient of the negative ion.

Not only the change in ion density on the gate electrode opening and on the channel surface can explain current gain for different solutions, but also voltage drop or potential drop can explain the drain current gain. From the point of view of FET operation, explanation with voltage drop for EDL FETs might be more intuitive and favored. When V_g_ is applied to the gate electrode, the gate voltage drops through the solution (Δ*V*_*s*_) and across the dielectric of the HEMT (Δ*V*_*ox*_) (Fig. 1(j)). Because our HEMTs are depletion mode FETs, which have very high conductivity in the active channel, we can ignore the drop of V_g_ in the channel to simplify the model, and assume that the V_g_ only drops across the solution and the dielectric, and then it becomes grounded in the channel with the source terminal, when V_d_ is applied to the drain terminal. By assuming that, the voltage drop of V_g_ can be divided as the voltage drop in solution, Δ*V*_*s*_, and the voltage drop in the dielectric, Δ*V*_*ox*_, as shown as in equation 5. We assume that in the solution, when V_g_ is applied, the capacitance of the solution *C*_*s*_ is generated due to the formation of EDL (Fig. 1(j)). By taking the advantage of the linear relationship between voltage drop and impedance, the voltage drop in dielectric can be shown as in equation 6, which indicates larger solution capacitance leads to larger effective V_g_ applied on the dielectric of the FET, thus causing larger drain current increase. For the application of biosensors, the detection of this FET-based sensor is based on the change of the solution capacitance caused by target protein binding with the antibody on the gate electrode opening. Of course, the immobilization of the antibody or aptamer, can also be monitored by the change of *C*_*s*_, leading to the change of the drain current gain. We can also conclude that in higher ionic strength solution, the current gain is larger due to the larger solution capacitance, which is consistent with the previous explanation with ion density in EDL. Significance of *C*_*s*_ in physics will be discussed in detail latter.

5 $${V}_{g}={\rm{\Delta }}{V}_{s}+{\rm{\Delta }}{V}_{ox}$$

6 $${\rm{\Delta }}{V}_{ox}=\frac{\frac{1}{j\omega {C}_{ox}}}{\frac{1}{j\omega {C}_{ox}}+\frac{1}{j\omega {C}_{s}}}\times {V}_{g}=\frac{{C}_{s}}{{C}_{ox}+{C}_{s}}\times {V}_{g}$$

where *j* and *ω* are current and angular frequency, respectively. Δ*V*_*s*_ and Δ*V*_*ox*_ are the gate voltage drops in solution and in dielectric, respectively. *C*_*s*_ and *C*_*ox*_ are the solution capacitance and the dielectric capacitance, respectively.

### EDL FET biosensors for HIV-1 RT and CEA detection in 1X PBS

Since we have known the fundamental properties and the principles of our FET biosensors, here we would like to show that these sensors can effectively detect proteins in physiological solution with high ionic strength. The first example is a single-stranded DNA (ssDNA) that is used as an aptamer, which is specific against HIV-1 RT and immobilized on the gate electrode opening through sulfur-gold bond. This aptamer was selected by Schneider *et al*. in 1995^20^. It has 35 mers and the active binding region is from the 8^th^ base from 5′ end to the 30^th^ base, according to the paper^20^. In order to immobilize the aptamer to our gold gate electrode, we add additional poly-dT with a thiol group in the 5′ end. The extended sequence is to allow the aptamer to have sufficient space to form secondary structure and bind to the HIV-1 RT. The distance from the gate surface to the active binding region of the aptamer is about 15 mers, which is about 4.9 nm. Although this number may not be the exact distance, it is reasonable to estimate the distance ~3 nm at least.

**Figure 2:**
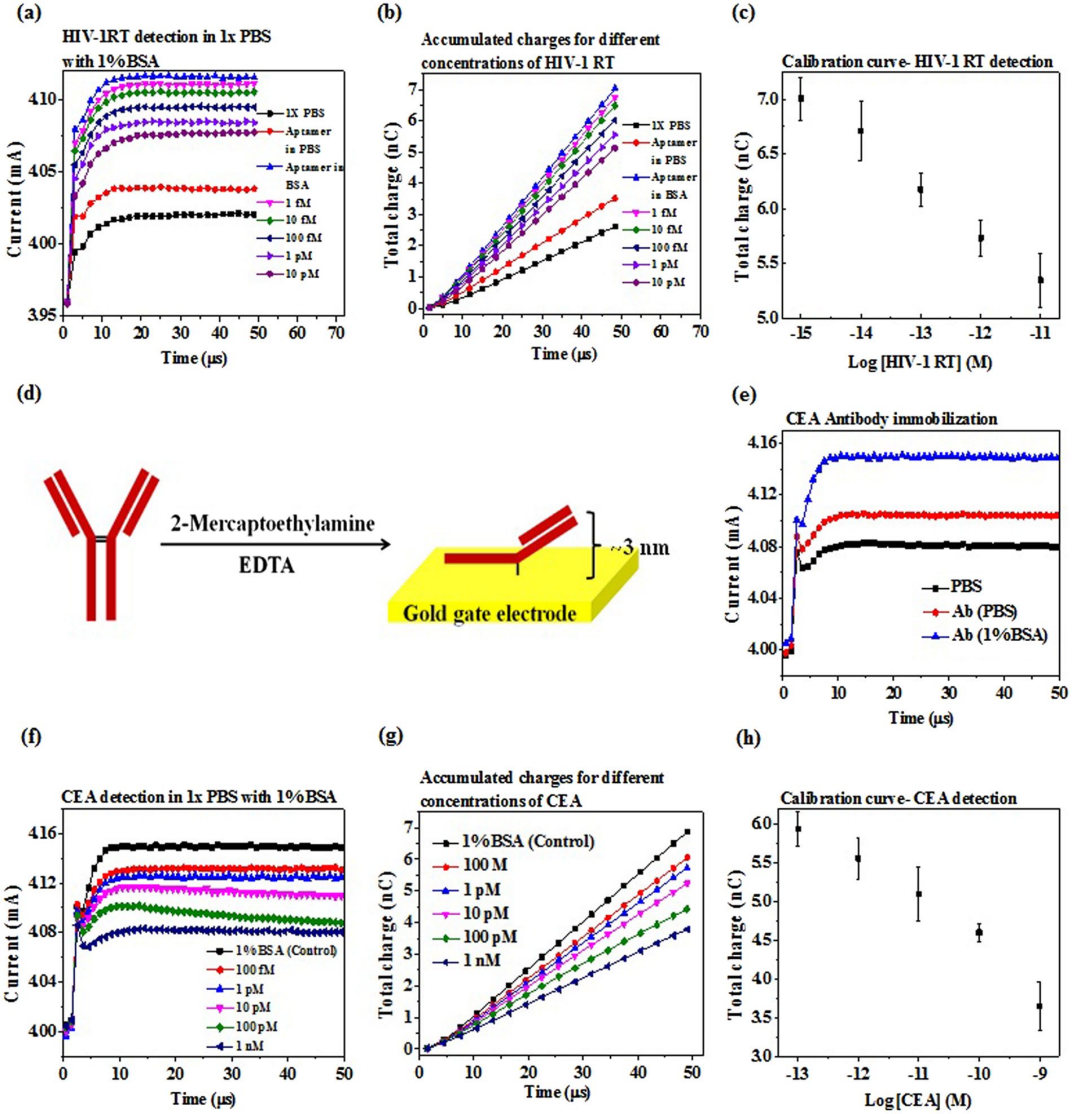
EDL FET biosensor for HIV1-RT and CEA detection. Purified samples are prepared in 1X PBS with 1% BSA in order to simulate the near real conditions of human serum. (**a**) Aptamer based HIV1-RT detection in 1X PBS containing 1% BSA. Thiolated ssDNA aptamer to HIV1-RT is immobilized on gold gate electrode opening via S-Au self-assembly. The output drain current vs. time graph show successful aptamer immobilization and concentration dependent current gain decrease. (**b**) Accumulated charges for each HIV1-RT concentration is calculated by integrating drain current over time when the gate bias is applied. Indexing total charge reduces the noise fluctuations and leads to better signal separation. (**c**) Calibration curve for HIV1-RT detection is plotted by measuring the total charge accumulated for each HIV1-RT concentration. The measured HIV1-RT concentrations are 1 fM, 10 fM, 100 fM, 1 pM and 10 pM. (**d**) Antibody immobilization technique. Antibody ‘hinge’ region’s disulfide bond is cleaved using mild reducing agent and the resulting native thiol groups of the half IgGs bind to gold via S-Au self-assembly. The protein binding site is nearly 3 nm away from the gold surface. (**e**) Successful antibody immobilization (anti-CEA) is depicted by the time domain drain current gain before and after immobilization. Since purified CEA proteins are prepared in 1X PBS with 1% BSA, device baseline is measured after anti-CEA immobilization in 1% BSA. (**f**) CEA concentration dependent drain current decrease. (**g**) Accumulated charges for each CEA concentration vs. time. (**h**) CEA calibration curve. The concentration dynamic range represents the clinically relevant CEA detection range. The measured CEA concentrations are 100 fM, 1 pM, 10 pM, 100 pM and 1 nM.

The HEMT was measured in time domain as previously at V_d_ = 2 V and V_g_ switching from 0 V to 0.5 V, with and without aptamer immobilization in 1X PBS (Fig. 2(a)). Here, the drain current gain is defined as at fixed V_d_, the drain current change caused by switch V_g_ = 0 V to V_g_ = 0.5 V. It is obvious that the aptamer cause the drain current gain increase more than 20 µA, compared to the bare HEMT. If 1% BSA is added into 1X PBS, the current gain will largely increase (Fig. 2(a)). Different HIV-1 RT concentrations are prepared in 1X PBS with 1% BSA solution and detected with the aptamer-immobilized HEMT. The HIV-1 RT solution is placed on the top of the sensor for 5 minutes, and then the current is measured, without any washing process. Compared to the 1X PBS with 1% BSA, the higher concentration of HIV-1 RT solution causes more current gain decrease. In order to get larger separation between different concentrations and to eliminate random noise caused by the device, the current gain is integrated with time as total charges (Fig. 2(b)). The experiments are repeated several times and the total charge versus HIV-1 RT concentration is plotted as the calibration curve for the sensor (Fig. 2(c)). The same sensor is washed with protein elution solution to remove HIV-1 RT from aptamer for repeated measurements. The elution time is optimized by washing the highest concentration of HIV-1 RT solution (10pM) after detection (Supplementary Fig. S1).

Another test is CEA detection based on antibody immobilized on HEMTs. 2-Mercaptoethylamine (MEA) was used to cleave the disulfide bond in heavy chain of the IgG antibody. The cleaved thiol group then bind on gold surface, leading to an orientated antibody, which leaves the binding site towards the upside^21^, and shortened distance between the binding site and the gate electrode. Although the distance between the binding site and the electrode surface is shortened, there still exists ~3 nm length in the gap (Fig. 2(d)). The gap is much larger than the Debye length in 1X PBS (0.7 nm). Immobilization of antibody causes current gain increase in 1X PBS, and 1% BSA further increase the current gain (Fig. 2(e)). Different CEA concentrations, ranging from 100 fM to 1 nM, cause decreasing current gain from the level of BSA solution. All the CEA solutions are prepared in 1% BSA in 1X PBS. Therefore, the current gain of the BSA solution becomes the background for the CEA detection (Fig. 2(f)). The total charge versus CEA concentration shows the same trend as the current gain (Fig. 2(g)). The calibration curve is established according to the total charge calculated at 50 µs for different CEA concentrations (Fig. 2(h)). The sensor is also washed with protein elution solution for repeated detections, and the elution process is confirmed by detecting CEA from low to high concentration, and vice versa. Both ways show the same trend on the electrical response (total charge vs. CEA concentration), demonstrating that the detection or sensitivity is not resulted from accumulation of remaining protein from previous tests (Supplementary Fig. S2).

In these two cases, both aptamer and antibody cause current gain increase from the bare HEMT, and the addition of BSA increases the current gain from aptamer or antibody in 1X PBS. Besides, both HIV-1 RT and CEA binding cause current gain decrease from the level of BSA.

### EDL FET biosensors for NT-proBNP detection in 1X PBS and human serum

**Figure 3:**
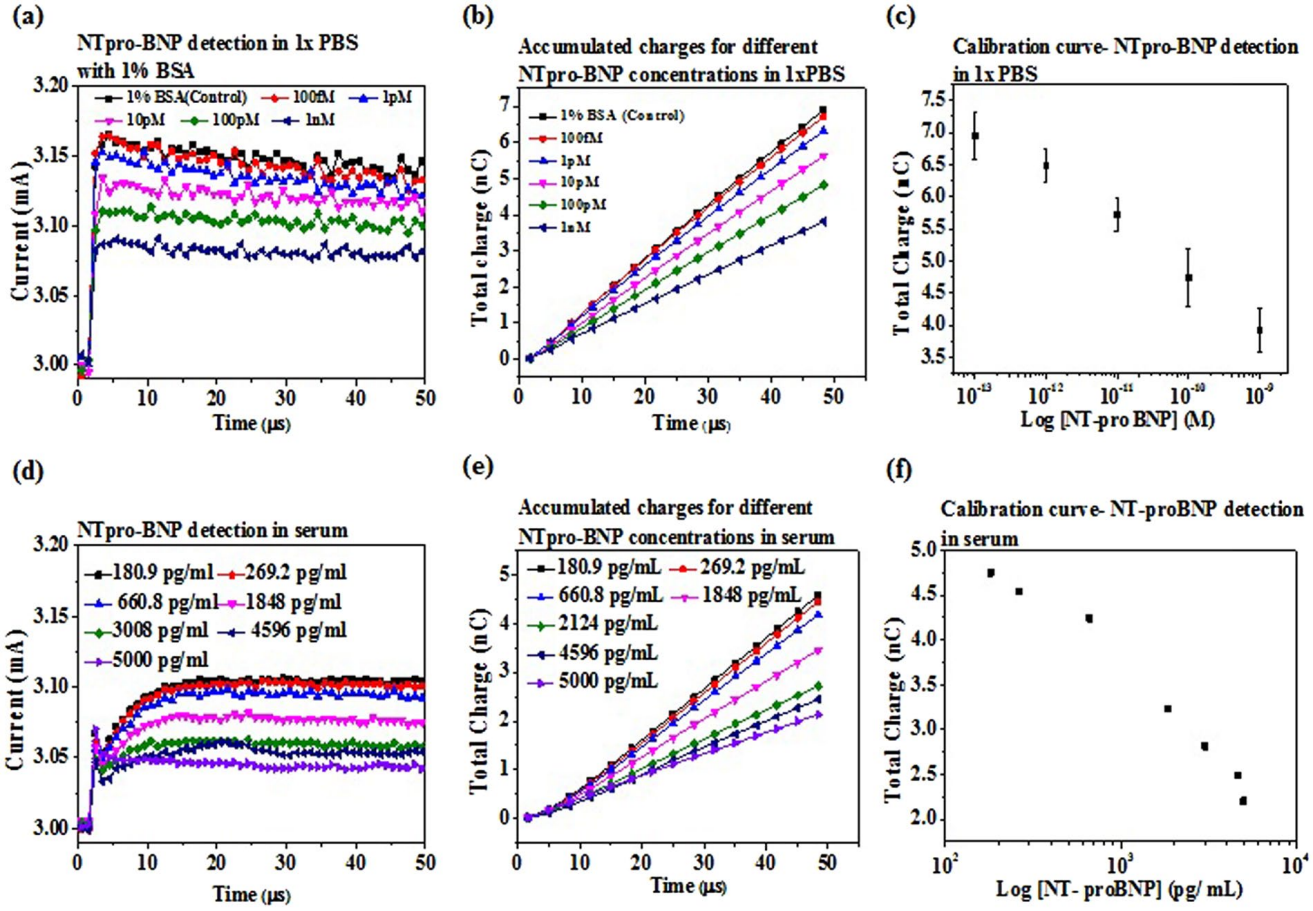
EDL FET biosensor for NT-proBNP detection. Tests are carried out using purified proteins prepared in 1X PBS with 1% BSA and clinical human serum. (**a**) Drain current vs time graph for NT-proBNP detection in 1X PBS with 1% BSA. Anti-NT-proBNP immobilization for the corresponding device is shown in Supplementary Fig. S3. (**b**) Accumulated charges calculated for each NT-proBNP concentration is plotted against time. (**c**) Calibration curve for NT-proBNP showing concentration dependent decrease in total charge. The measured NT-proBNP concentrations are 100 fM, 1 pM, 10 pM, 100 pM and 1 nM. (**d**) Drain current in time domain is depicted for several concentrations of NT-proBNP in clinical human serum. Anti-NT-proBNP immobilization for the corresponding device is shown in Supplementary Fig. S4. (**e**) Total charge accumulated plotted against time for different NT-proBNP concentrations. (**f**) Calibration curve for NT-proBNP detection in human serum with total charge vs. concentration. The measured NT-proBNP concentrations are 180.9 pg/mL, 269.2 pg/mL, 660.8 pg/mL, 1848 pg/mL, 3008 pg/mL, 4596 pg/mL and 5000 pg/mL.

Immobilization of NT-proBNP antibody is confirmed by comparing the current gain of the bare HEMT with that of the antibody-immobilized HEMT in 1X PBS (Supplementary Fig. S3). The antibody significantly increases the current gain. The current gain is measured in time domain for 1% BSA in 1X PBS, and NT-proBNP solutions ranging from 100 fM to 1 nM in 1X PBS with 1% BSA (Fig. 3(a)) The integrated total charge within 50 µs for different NT-proBNP shows same trend as the current gain (Fig. 3(b)). The total charge at 50 µs for each NT-proBNP concentration is established as calibration curve (Fig. 3(c)). The addition of BSA in 1X PBS causes current gain increase and the binding of NT-proBNP with antibody decrease the current gain (Fig. 3(c)). Successful protein elution process is also confirmed (Supplementary Fig. S4).

Since NT-proBNP detection with the antibody-immobilized HEMT in 1X PBS with 1% BSA works well, we therefore directly test NT-proBNP in 7 human serum samples. Antibody immobilization is confirmed first (Supplementary S5). The concentrations of NT-proBNP in human sera, including 180.9 pg/ml, 269.2 pg/ml, 660.8 pg/ml, 1848 pg/ml, 3008 pg/ml, 4596 pg/ml, and 5000 pg/ml, are tested with the sensor and show decreasing current gain with increasing concentration of NT-proBNP (Fig. 3(d)). The integrated total charge and the calibration curve show very successful detection in human sera (Fig. 3(e,f)). More importantly, although these human sera are obtained from different patients, they show very good linearity, which indicates the same electrical background responses are obtained from these sera. The values of the NT-proBNP concentrations are measured with Beckman system used in hospital. The results from human serum show that non-specific binding is not an issue for our sensor. The detection of NT-proBNP with the sensor shows the same trend in buffer solution and in human serum.

### EDL FET biosensors for CRP detection in 1XPBS and human serum

**Figure 4:**
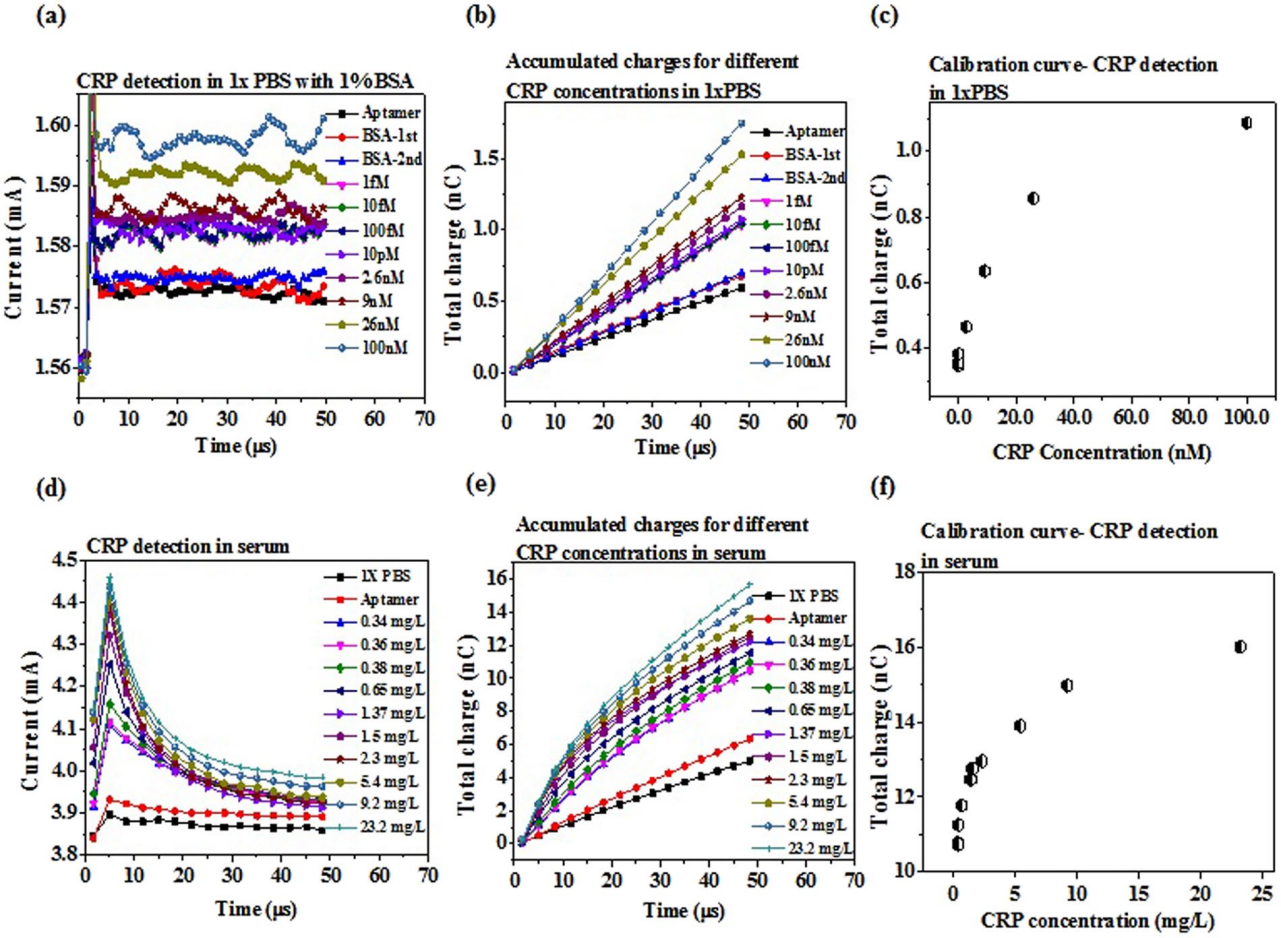
EDL FET Biosensors for CRP detection. Purified proteins prepared in 1X PBS with 1% BSA and clinical human sera are tested using HEMT integrated with microfluidic device. (**a**) Aptamer based detection in time domain for CRP prepared in 1X PBS with1% BSA. Concentration dependent drain current increase is observed. (**b**) Accumulated charges vs. time for each CRP concetration. Signal to noise ratio is improved by using total charge as the index as random noise is cancelled out. (**c**) Calibration curve for CRP detection in 1X PBS with 1% BSA. The measured CRP concentrations are 1 fM, 10 fM, 100 fM, 10 pM, 2.6 nM, 9 nM, 26 nM and 100 pM. (**d**) CRP detection in time domain for clinical human serum samples. (**e**) Accumulated charges for different CRP concentrations in serum. (**f**) Calibration curve for CRP detection in serum. The measured CRP concentrations are 0.34 mg/L, 0.36 mg/L, 0.38 mg/L, 0.65 mg/L, 1.37 mg/L, 1.5 mg/L, 2.3 mg/L, 5.4 mg/L 9.2 mg/L 23.2 mg/L.

CRP is detected with an aptamer-immobilized HEMT integrated with a microfludic channel in 1X PBS and in human serum. The aptamer has been proved with high affinity and high specificity previously^22, 23^. The aptamer has 72 bases and the 5′ end is attached with a thiol group. The distance between the binding site and the electrode surface is estimated as at least 3 nm, based on the potential secondary structure of the aptamer. Different CRP concentrations, including 1 fM, 10 fM, 100 fM, 10 pM, 2.6 nM, 9 nM, 26 nM, and 100 nM are prepared in 1X PBS with 1% BSA and tested with similar procedures, such as the current gain in time domain, integrated total charge, and the calibration curve (Fig. 4(a,b,c)). In such high ionic strength solution, the sensor shows excellent sensitivity. CRP is a pentameric protein and has a molecular weight ~125 kDa. Therefore 9 nM and 26 nM are nearly 1 mg/L and 3 mg/L, respectively. For CVD risk evaluation, patient with CRP level less than 1 mg/L is regarded as at low risk. When the CRP level is within 1~3 mg/L, patient will be regarded as at middle risk. If the level rises beyond 3 mg/L, the risk for CVD is high^24^. This is the range where the high sensitivity CRP (hsCRP) is defined. Our sensor shows extremely high sensitivity in 1X PBS with 1% BSA.

10 human sera are then tested for CRP in hospital with Beckman system and with our sensor. The concentration of CRP of the 10 human sera measured in hospital includes 0.34 mg/L, 0.36 mg/L, 0.38 mg/L, 0.65 mg/L, 1.37 mg/L, 1.5 mg/L, 2.3 mg/L, 5.4 mg/L, 9.3 mg/L, and 23.2 mg/L. Our sensors can effectively distinguish these sera, showing the high sensitivity once again. The aptamer causes current gain increase in both 1XPBS and human serum, which show the same trend as the aptamer against the HIV-1 RT. However, CRP binding with aptamer causes current gain increase in both 1X PBS and human serum, which shows different trend from HIV-1 RT, CEA, and NT-proBNP binding with aptamer or antibody.

### Mechanism of EDL FET biosensors

We have shown that four different proteins can be effectively detected with our FET biosensors in high ionic strength solution (1X PBS containing 1% BSA) and in real clinical samples, without any dilution. Aptamers against HIV-1 RT and CRP both cause current gain increase in 1X PBS. Antibody against CEA and NT-proBNP also cause current gain increase in 1X PBS. NT-proBNP detection in 1X PBS and serum both decrease current gain with increasing NT-proBNP concentration. CRP detection in 1X PBS and serum both decrease the current gain with increasing CRP concentration. All the protein detections form excellent quantitative calibration curve. These results are consistent and repeated, which strongly demonstrate the reliability and capability of our FET biosensors for direct protein detection in physiological environment beyond the Debye length. We have shown that the electrical response of the sensor is explained with the variation in voltage drop caused by the protein binding, as shown in equation 5 and 6. However, what is the physics behind the electrical response? Why can our sensor effectively detect protein beyond Debye length? What is the model for this sensor? These questions are what we would like to answer in this section.

We know that Grahame equation, as shown in equation 7, is frequently used to show the relationship between surface potential and surface charge density on a planar surface, either on metal or non-metal surface^25^.

7 $$\sigma ={(8n{\varepsilon }_{r}{\varepsilon }_{0}kT)}^{1/2}\,\sinh (\frac{ze{\psi }_{0}}{2kT})$$

where *σ*, *ψ*_0_, *ε*_*r*_, *ε*_0_ and *n* are surface charge density, surface potential, relative permittivity, permittivity in vacuum, and electrolyte concentration in bulk solution, respectively.

In this equation, we can easily see that larger surface potential can generate higher charge density. In our EDL HEMT, when larger V_g_ is applied on the gate electrode, higher current gain is obtained, which can be explained with larger voltage drop in dielectric, or more charges accumulated on the gate electrode opening, leading to equal counter charge accumulated on the channel surface. It seems that the behavior of our EDL HEMT can be explained with Grahame equation. However, we know that the current gain of our EDL HEMT does not only depend on the V_g_, but also on the gap between the gate electrode and the channel (Fig. 1(h,i)). It is obvious that in Grahame equation, the charge density is not relevant to the gap between the gate electrode and the channel. If we go back to check the origin of the Grahame equation, we can find that it is the solution of a non-linear Poisson-Boltzmann equation, as shown in equation 8.

8 $$\frac{{d}^{2}\psi (x)}{d{x}^{2}}=-\frac{\rho (x)}{{\varepsilon }_{r}{\varepsilon }_{0}}=\frac{2zen}{{\varepsilon }_{r}{\varepsilon }_{0}}\,\sinh (\frac{ze\psi (x)}{kT})$$

The boundary conditions for this equation are shown as followings,

9 $$\psi ={\psi }_{0}\,{\rm{at}}\,x={\rm{0}}$$

10 $$\psi =\frac{d\psi }{dx}=0\,{\rm{at}}\,x=\infty $$

**Figure 5:**
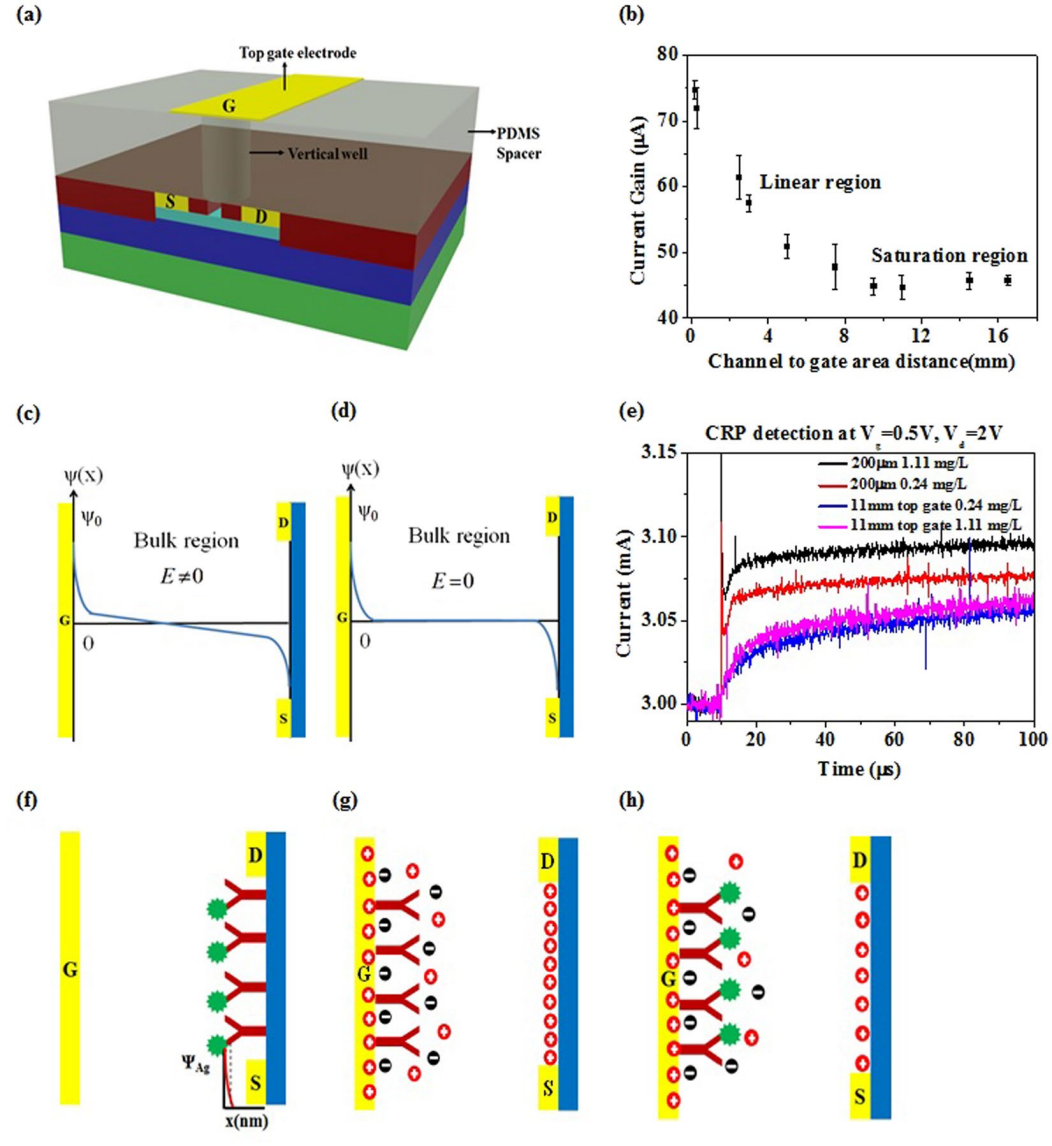
Working mechanisms of EDL FET biosensors. (**a**) Schematic representation of the experiment to determine the dependence of voltage drop and hence drain current on a large range of distances between gate opening and active channel. Gate electrode is vertically positioned on top of the active channel separated by the PDMS spacer of varying heights. Typical gate electrode opening area is used in this experiment. Thus the gap distance between gate opening and active channel are designed as 200 µm, 300 µm, 2.5 mm, 3 mm, 5 mm, 7.5 mm, 9.5 mm, 11 mm, 14.5 mm and 16.5 mm. (**b**) Calculated current gain vs. active channel to gate opening area distance graph. A linear relationship is obtained for smaller distances and saturation is observed for larger distances where the current gain is independent of the distance. (**c**) Our sensor is designed to operate in the denoted linear region and the applied gate voltage creates a potential gradient across the solution. (**d**) Depiction of saturation region where the potential gradient is only near the electrode surface. (**e**) Detection of CRP in linear region and saturation region of gap distances. 0.24 mg/L and 1.11 mg/L of CRP serum samples are tested. Smaller gap leads to larger separation and larger gap lead to very small separation. (**f**) Conventional FET biosensor schematic model. Charge of the target protein modulates the conductivity of FET. (**g**) Sensing mechanism in our sensor Distribution of charge density prior to the introduction of target proteins. (**h**) Protein binding induced gate voltage drop causes local charge density re-distribution in the gate electrode area leading to subsequent changes in the charge density on the active channel.

The boundary condition as shown in equation 10 determines the solution of the Poisson-Boltzmann equation. In our sensor, the gap between the gate electrode and the channel is limited. If the gap is really large, the boundary condition in equation 10 may work. If it is not the case, then this boundary condition may not be appropriate for our sensor. How do we know in what dimension the gap is appropriate or not for that boundary condition? The most straightforward way is to measure our EDL HEMT from small gap to very large gap, and see how the current gain, which represents the voltage drop in dielectric, will vary with the gap in a large range of dimension. To do that, we designed a vertically displaced gate electrode, using the typical active channel and the same gate electrode opening as previously (Fig. 5(a)). The gap between the vertical gate electrode and the active channel includes 200 µm, 300 µm, 2.5 mm, 3 mm, 5 mm, 7.5 mm, 9.5 mm, 11 mm, 14.5 mm, and 16.5 mm. The current gain is measured at V_d_ = 2 V and V_g_ = 0.5 V. The current gain and the gap show two different relations, including linear dependence (linear region) and independence (saturation region) (Fig. 5(b)). When the gap is larger than 9.5 mm, the current gain keeps the same. When the gap is smaller than 7.5 mm, the current gain shows linear relationship with the gap. Between 7.5 mm and 9.5 mm, it is more likely a transition region between linear and saturation regions. In the saturation region, there is considerable current gain, indicating the voltage drop across the dielectric and in the solution as well, which means that the Grahame equation actually works in this saturation region. On the other hand, in the linear region, the linearity of the current gain actually indicates that the voltage drop in solution is also linearly dependent on the gap, which means that the solution capacitance (*C*_*s*_) behaves as a conventional solid capacitor. Voltage drop in a solid capacitor is linearly decreased with the thickness of the capacitor, resulting in a uniform electric field in the capacitor. Thus, the V_g_ drop in solution also keeps the potential gradient through the solution (Fig. 5(c)). In the bulk solution, the potential gradient still exists, which means the electric field in the bulk solution is not zero. This is actually similar to the environment as in electrophoresis^26–28^, in which an electric field is at least larger than 1 V/cm. In our sensor, the typical gap is around 265 µm and the V_g_ is around 0.5 V. For simplicity, if we ignore the voltage drop in dielectric, then all the V_g_ drops in solution and the field we apply across the solution is around ~20 V/cm. If the criterion of the field is 1 V/cm, then we can predict that when our gap is larger than 5 mm, the current gain will no longer be linearly dependent on the gap. This prediction quite fits to our experimental results in the magnitude of the order. For the saturation region, the potential gradient only exists near electrode surface, and in the bulk solution region, the potential is zero or constant and the electric field does not exist (Fig. 5(d)).

Now, we need to explain why our sensor can detect protein beyond Debye length. The EDL HEMT with the vertically displaced gate electrode is immobilized with CRP aptamer, and is measured with the current gains to detect two concentrations of CRP, including 0.24 mg/L and 1.11 mg/L, in human serum with two different gaps, 200 µm and 11 mm, respectively (Fig. 5(e)). It is obvious that the smaller gap (200 um) leads to a significantly larger separation between the two concentrations (Fig. 5(e)). However, the larger gap (11 mm) results in extremely tiny separation (Fig. 5(e)). The high sensitivity obtained in the smaller gap, which is in the linear region is attributed to the extended potential gradient through the solution, leading to the effective protein detection beyond Debye length (Fig. 5(c)). This can be explained as the protein located outside Debye length can still sense the field in the solution when the gap is in the linear region (Fig. 5(c)). In sharp contrast, the protein outside Debye length in the saturation region cannot feel any potential or electric field, thus resulting in difficulty in the detection in high ionic strength solution (Fig. 5(d)).

In the conventional FET biosensor’s theory, the detection is based on the charge of the target protein, which results in a potential change near the surface of the channel, leading to the current change of the FET (Fig. 5(f)). Because the detection relies on the charge of the protein or the potential created by the protein, the target protein has to be as close to the channel surface as possible to create larger potential on the active channel and obtain better sensitivity. In our sensors, since proteins are captured on the gate electrode, which is 65~265 µm away from the channel, it is not possible to rely on the charge of the protein or the potential of the protein that delivers to the channel surface across such huge gaps and change the conductivity of the FET. Instead, our sensor rely on the effective gate voltage drop caused by protein binding, which re-distributes the charge density on the gate electrode and the channel surface as well due to the neutrality of the total change in solution (Fig. 5(g,h)), thus leading to the detection of the protein.

**Figure 6:**
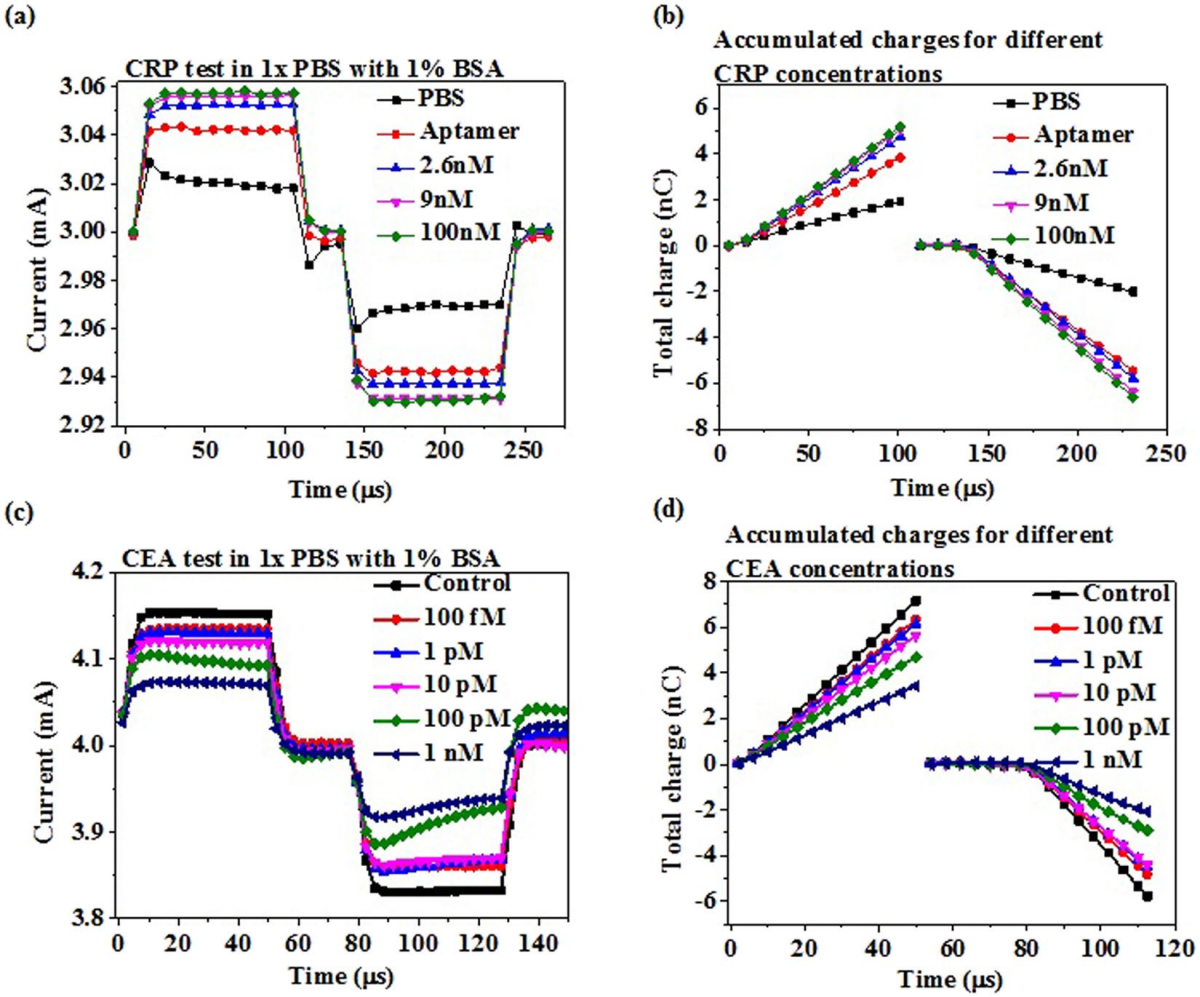
Comparison of sensor response in dual gate bias. Samples prepared in 1x PBS with 1% BSA are used for the experiment. (**a**) CRP detection in time domain for 2.6 nM, 9 nM and 100 nM. (**b**) Accumulated charges for each CRP concentration. The symmetric nature of signal in both positive and negative bias validates the proposed FET biosensor model. (**c**) CEA detection in time domain for 100 fM, 1 pM, 10 pM, 100 pM, 1 nM. (**d**) Accumulated charges for each CEA concentration. Symmetry is observed as in the case of (**a**) (**b**) for CEA as well.

Also, in conventional FET biosensor’s theory, the sign of the net charge of the target protein determines whether the protein increase or decrease the surface potential on the channel^29^. Do the sign of the net charge of the target protein also dominate the detection of our sensors? The isoelectric points (*pI*s) of HIV-1 RT, CEA, NT-proBNP, and CRP are reported as 8~9^30^, 4.7^31^, 8.5^32^, and 7.4^33^, respectively. From our results, HIV-1 RT, CEA, and NT-proBNP cause current gain to drop from baseline as their concentration increase, while CRP causes current gain increase (Figs 2, 3 and 4). The results do not show any relevance of the increasing or decreasing trend to the isoelectric points. The trend in 1X PBS is the same as in the human serum. To doubly confirm that the sign of the net charge of target protein is not the key in the detection for our sensors, we compare our results measured with positive and negative gate bias at the same drain bias (Fig. 6). CRP is tested with positive and negative V_g_ (Fig. 6(a)) and the total charges are counted for several CRP concentrations. The current gains and the total charges in positive V_g_ and negative V_g_ are nearly symmetric, showing that higher concentration of CRP cause more current gain increase in positive V_g_ or decrease in negative V_g_ (Fig. 6(a,b)). Similar symmetric curves are also obtained for CEA detection, which shows that higher CEA concentration cause more current gain drop in positive V_g_ and more current gain increase in negative V_g_ (Fig. 6(c,d)). If we assume that CRP brings negative chargers at 1X PBS, thus resulting in the increased negative charge density near the gate electrode surface in solution at positive V_g_, leading to increased current gain as the CRP concentration increases, then we should expect that at negative V_g_, CRP should decrease positive charge density near the gate electrode surface, causing smaller gain decrease (less negative current gain), leading to un-symmetric trend of CRP concentrations. However, for both CRP and CEA detection with our sensors, we get all nearly symmetric curves. Here the “symmetric curve” is defined as the trend of current gains or charges caused by different protein concentrations, but not the exact value of the current gains or charges. These results demonstrate that the detection of our sensors do not depend on the sign of the net charge of the target proteins, which are different from conventional FET biosensor’s theory.

Then, what is the mechanism of the detection of our sensors if it is not dependent on the sign of the net charge of the protein? We have learned that higher ionic strength can cause larger current gain (Fig. 1(c)). The detection of our sensors is attributed to the increase or decrease of ionic strength in local region caused by aptamer or antibody, and the binding proteins, including HIV-1 RT, CEA, NT-proBNP, and CRP, as well. The charge density of double stranded DNA (dsDNA) was reported at least one order of magnitude higher than physiological salt concentration^34, 35^. For aptamers, although we may not be able to precisely predict their charge density, it is reasonable to assume that their charge density is in the same order of magnitude as dsDNA. That can explain why HIV-1 RT aptamer and CRP aptamer both cause current gain increase. Because the detection of our sensors is attributed to the ionic strength, the net charge or the sign of the charge of protein does not matter. The change of ionic strength in local region can be resulted from the valence, the amount of dissociable functional groups of proteins or aptamers, and the stereo-hindrance caused by proteins. It may also result from the bending or moving of the aptamers or proteins due to the electric field in solution. Further investigation in detail will be needed and may be done by simulation of molecular dynamics, which is beyond the extent of this study.

Could the pulse-biased signal, which is the current gain, not be at steady state due to the short duration of the pulsed signals? To verify that, we investigated different pulse times, including 20 µs, 50 µs, and 1 ms. The results show all the three bias conditions give the same steady current gain (Supplementary Fig. S6). We therefore confirm that our sensor does work in the steady state and the signals extracted as well.

## Discussion

As previously mentioned, the conventional FET biosensor’s theory is based on the potential created by the target protein itself that delivers to the channel surface to modulate the FET. In order to deliver larger potential, the solution is usually diluted from 10~10,000 times^6, 7^, to reduce the charge screening effect, leading to much lower ionic strength than that in physiological samples. The dilution is not only inconvenient in handling with physiological samples, but also risky due to the possible loss of the protein activity caused by structural change of protein in low ionic strength solution. In fact, our sensors generate larger signals in high ionic strength solution that that in low ionic ones. The method of our sensors is particular suitable for physiological samples than the diluted ones. The signal of our sensor is based on the current gain, which is larger in high ionic strength solution than in low ionic strength one. When the sensor is operated in higher current gain, the separation among different concentrations of target protein is amplified, resulting in higher sensitivity. Our sensor turns the “drawback”, which is high ionic strength for conventional FET biosensors, into the “advantage”, which gives high sensitivity for our sensors. Another important feature we have observed in our sensor is that when V_g_ increases, the current gain also increases, which leads to the increase of the signal. More importantly, the noise keeps almost the same when V_g_ increases. This represents that the high current gain of our sensor can increases the signal-to-noise (S/N) ratio.

If we look back to the results of serum samples, including NT-proBNP and CRP, we quickly find that their calibration curves look pretty well (Figs 3(f) and 4(f)). The calibration curves in 1X PBS and in serum show the same trend. Although the human sera are from different patients, it seems that the calibration curves are just dependent on the target protein concentration and show very consistent data in the calibration curve. Large deviations from the calibration curve among patient’s samples are not found. This result indicates that these patients’ sera give same electrical background and that is why the curve only looks dependent on the target protein concentration. To verify that idea, we test several sera from different patients with the same HEMT device, which is not immobilized with aptamer or antibody. The HEMT is measured once the sera are dropped and protein elution process is conducted as the same as the typical elution process described previously. The current gains for all these serum samples are almost the same, which identify the same background response for the FET for different sera (Supplementary Fig. S7). This is probably that the ionic strength and major composition in most people’s blood are nearly the same. Even though people may have different specific protein concentration, the summation of all the proteins, metabolites and salts make the overall electrical response become averaged and the same for most people. This interesting result proves that the sensor can detect target protein directly in different patient’s serum without additional washing process.

We have mentioned several advantages of our sensors previously. There are still some other advantages worth to mention. For example, because our detection does not rely on the charge of the target protein, our sensors can detect charged or un-charged proteins. This feature allows our sensor to detect all kinds of proteins. Also, our sensor is measured with a single pulse in a short duration of 50 µs, which largely reduces the heat generated by the HEMT, thus reducing the thermal noise, leading to great repeatability. Furthermore, the signal of the sensor is defined as “current gain”, but not the absolute drain current, which avoids the variation in serial resistance caused by the environment. The absolute drain current is easily affected by small fluctuation in environment, while the current gain is much more stable. Besides, the current gain is directly relevant to the solution capacitance, which is what we are interested. Finally, with the integration of current with time, the total charge is the sum of the current in unit time interval, which is mathematically close to the average of the current gains for each interval of unit time, resulting in elimination of the random noise, thus leading to a steady signal output for this sensor. With all these efforts done on this sensor, it has great potential to achieve direct protein detection in human sera with FETs with less pre-treatment, which is promising to point-of-care, homecare, and mobile diagnostic devices.

## Methods

### Fabrication of AlGaN/GaN High electron mobility transistors (HEMTs)

The AlGaN/GaN HEMT structure consists of 3 μm-thick undoped GaN buffer layer, 150 Å -thick undoped Al_0.25_Ga_0.75_N layer, and 10 Å -thick undoped GaN cap layer. The AlGaN/GaN epi wafer is deposited by molecular beam epitaxy (MBE) on silicon substrate. Device active region is defined by inductively coupled plasma (ICP) etching with Cl_2_/BCl_3_ gases (35 sccm/35 sccm) under ICP power of 300 W and RF bias of 120 W at 2 MHz. Ohmic contacts are fabricated by deposition of 200 Å -thick Ti layer, 400 Å -thick Al layer, 800 Å -thick Ni layer and 1000 Å -thick Au layer using electron beam evaporator, followed by rapid thermal annealing at 850 °C for 45 seconds in N2 environment. The ohmic contacts (60 × 60 μm^2^) of source and drain metals are separated by 30 μm gap and the transistor’s channel width is 50 μm. 1200 Å -thick Au layer forms the metal interconnects and the gate electrode. A passivation layer was generated by spin coating a positive 2 μm-thick photoresist (Shipley S1818), followed by a typical photolithography process to create the openings on the gate electrode and on the transistor channel. The gap between the openings on the gate electrode and on the transistor channel includes 65 μm, 265 μm and 465 μm. The devices are ready for surface modification and subsequent analyte detection.

### Sequence of the aptamer for HIV-1 RT and CRP

The sequence of HIV1-RT aptamer is: 5′-SH-TTT TTT TAT ACG TGA GCG TGC TGT CCC CTA AAG GTG ATA CGT CAG GGG-3′. The sequence of CRP aptamer is: 5′-GGC AGG AAG ACA AAC ACG ATG GGG GGG TAT GAT TTG ATG TGG TTG TTG CAT GAT CGT GGT CTG TGG TGC TGT-3′.

### Antibody and aptamer immobilization on HEMTs

Gold gate electrode is functionalized with receptors (antibody and aptamer) by the strong S-Au covalent binding. Disulfide bonds in the hinge region of IgG molecule is cleaved using moderate reducing agent such as 2-mercaptoethylamine (2-MEA) resulting in thiol terminated half IgG molecules which can self-assemble on to the gold gate electrode. Monoclonal antibodies (anti-CEA and anti-NT-pro BNP) are obtained from Abcam and 2-MEA from Sigma Aldrich. IgG and MEA mixture is incubated in room temperature on the device for 1.5 hours and at 4 °C for 12 hours. Unbound half-IgG is washed away using PBS before detection of proteins. Thiol modified oligonucleotides specific to HIV-1 RT and CRP are purchased from Genomics. Original aptamer solution is diluted in TE buffer containing tris (2-carboxyethyl)phosphine (TCEP) in the molar ratio 1:1000 and heated up to 95 °C to separate the single stranded DNAs. After the mixture is cooled down, 5 µl is dropped on the device and incubated at room temperature for 24 hours. The device is thoroughly washed in TE buffer and PBS prior to measurement.

### Protein samples in 1X PBS

For the detection of proteins in buffer system, all the proteins are diluted from their original concentrations in 1x PBS (137 mM NaCl, 2.7 mM KCl, 10 mM Na_2_HPO_4_, 2 mM KH_2_PO_4_, pH 7.4 with NaOH) containing 1% Bovine serum albumin (BSA) in order to simulate the physiological conditions of human blood that has high non-specific background protein concentration and high ionic strength. HIV-1 RT, CEA, NT-pro BNP proteins are purchased from Abcam and CRP from Sigma Aldrich.

### Human Serum samples

Clinical samples of human serum containing CRP and NT-pro BNP are used for electrical detection without further sample treatments. The samples are obtained as per Institutional Review Board (IRB) of National Cheng-Kung University Hospital (NCKUH) approval (IRB. No. B-ER-104-116) and under supervision by National Tsing Hua University IRB approval (10405HE014). All methods were carried out in accordance with relevant guidelines and regulations. All experimental protocols were approved by the IRB of NCKU and NTHU. Informed consents were waived according to IRB approval. The target analyte concentration in the clinical samples is determined using Beckman Coulter system. CRP concentration in serum samples are in the range of 0.34–23.2 mg/l and NT-pro BNP concentrations are in the range of 180.9–5000 pg/ml.

### Integrated microfluidic device

Detection of CRP in human serum samples has been carried out using AlGaN/GaN HEMT integrated with a microfluidic chip. The integrated microfluidic chip was composed of two polydimethylsiloxane (PDMS) layers including a liquid channel layer (thick-film PDMS layer) and a pneumatic layer (thin-film PDMS layer) bonded to a printed circuit board (PCB) using double-sided tape and the FET sensor. It was equipped with micropumps, microvalves and micromixers for automating the entire detection process.

### Sensors measurements

The source drain current characteristics are measured using Agilent B1530/B1500A semiconductor parameter analyzer system. 50 μs wide short duration pulse of 0.5 V is applied as gate voltage. The drain to source bias is fixed at 2 V. The sampling rate is 1 sample per 10 ns.

### Protein elution process

The sample containing target protein is placed on the sensor for 5 minutes to facilitate receptor-ligand binding followed by electrical detection. After each measurement, mild protein elution buffer solution (pH = 6.6) is used to elute the target proteins and non-specific binding proteins, if any, from the device surface. Device is alternatively washed and soaked for 45 minutes in 3 ml of elution buffer solution followed by washing in PBS and blow drying in air. Electrical measurements confirm the complete removal of proteins from FET surface.

## Electronic supplementary material

Supplementary information
