# Supplementary material for: Beyond the Debye length in high ionic strength solution: direct protein detection with field-effect transistors (FETs) in human serum
Source: Sci Rep. 2017 Jul 12;7:5256. doi: 10.1038/s41598-017-05426-6 (PMC5507911; doi:10.1038/s41598-017-05426-6)
Supplement: Supplementary file 1 — Supplementary information [file 41598_2017_5426_MOESM1_ESM.pdf]

Supplementary Information

Beyond the Debye length in high ionic strength solution: direct protein detection with field-effect transistors (FETs) in human serum

Chia-Ho Chu[1], Indu Sarangadharan[1], Abiral Regmi[1], Yen-Wen Chen[1], Chen-Pin Hsu[1], Wen-Hsin Chang[1], Geng-Yen Lee[4], Jen-Inn Chyi[4], Chih-Chen Chen[1,2], Shu-Chu Shiesh[5], Gwo-Bin Lee*[1,2,3] and Yu-Lin Wang*[1,2]

1. Institute of Nanoengineering and Microsystems, National Tsing Hua University, Hsinchu, 300, Taiwan, R.O.C.

2. Department of Power Mechanical Engineering, National Tsing Hua University, Hsinchu 300, Taiwan, R.O.C.

3. Institute of Biomedical Engineering, National Tsing Hua University, Hsinchu 300, Taiwan, R.O.C.

4. Department of Electrical engineering, National Central University, Jhongli City, Taoyuan County 320, Taiwan, R.O.C.

5. Department of Medical Laboratory Science and Biotechnology, National Cheng Kung University, Tainan City 701, Taiwan, R.O.C.

***Correspondence to ylwang@mx.nthu.edu.tw**

***Co-correspondence to gwobin@pme.nthu.edu.tw**

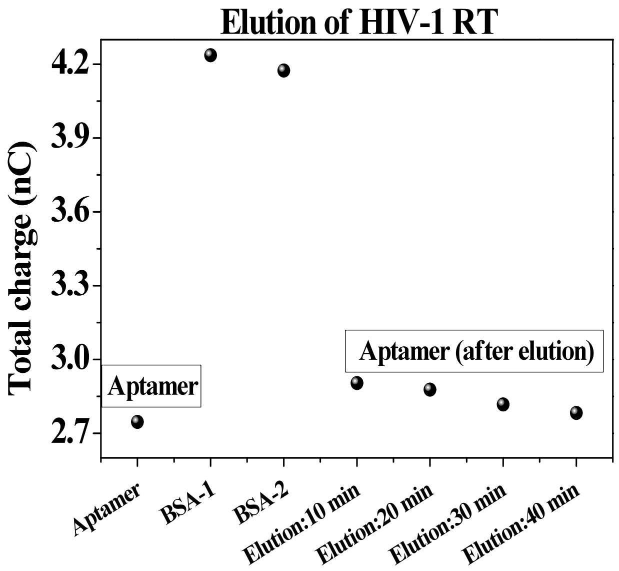

Figure S1 The process of elution of HIV1-RT from aptamer. Total charges corresponsing to aptamer before binding with target and after target elution keep close values, depicting successful protein elution. The elution process is carried out after each HIV1-RT test.

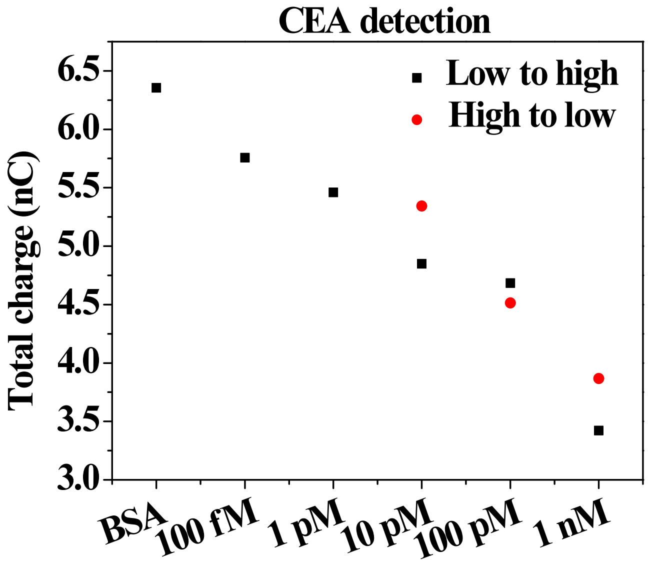

Figure S2 .CEA detection in 1X PBS with 1% BSA from low to high concentrations and vice versa. Elution process is used to wash away the bound CEA after each measurement. Increase in total charge when tested from high to low concentrations proves that the bound proteins and non specific proteins are washed away using the process of elution.

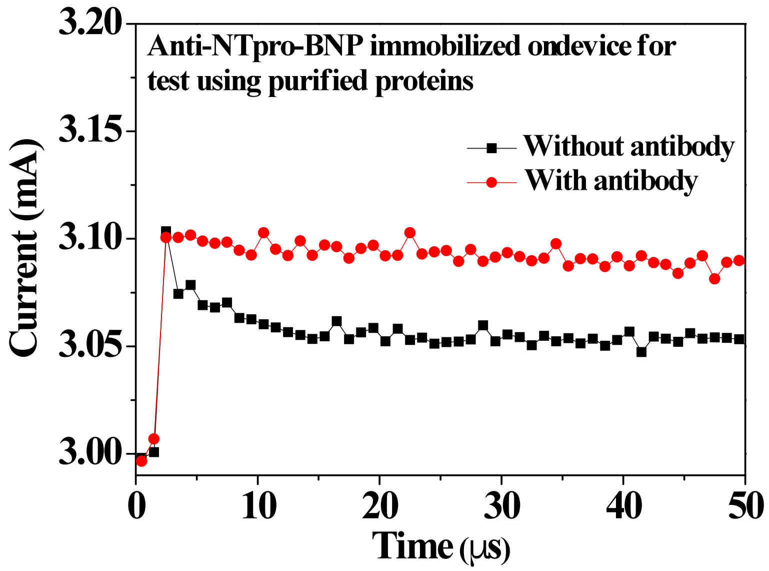

Figure S3 Successful immobilization of antibody to NT-proBNP on the device used for testing with purified proteins.

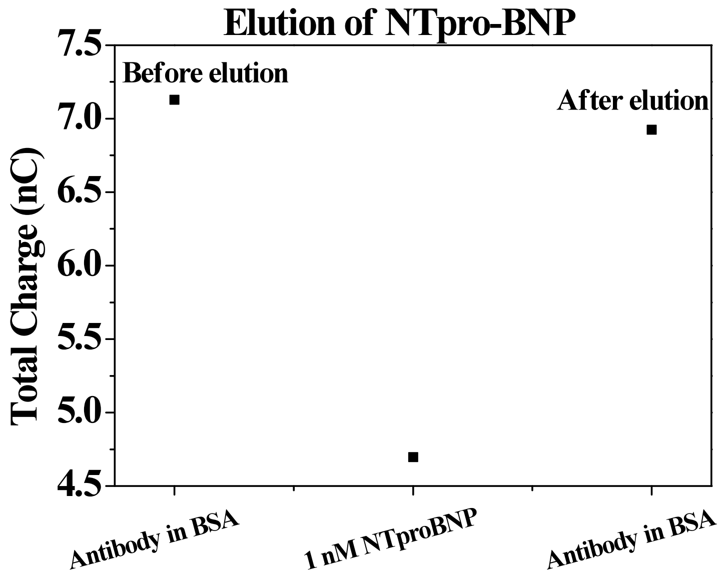

Figure S4 the process of elution of NT-proBNP from the antibody. Total charge corresponding to antibody measured in 1% BSA is close to the initial baseline of antibody in 1% BSA before the introduction of target protein (1 nM NT-proBNP). Very high concentration of target proteins can also be successfully eluted from the sensor surface.

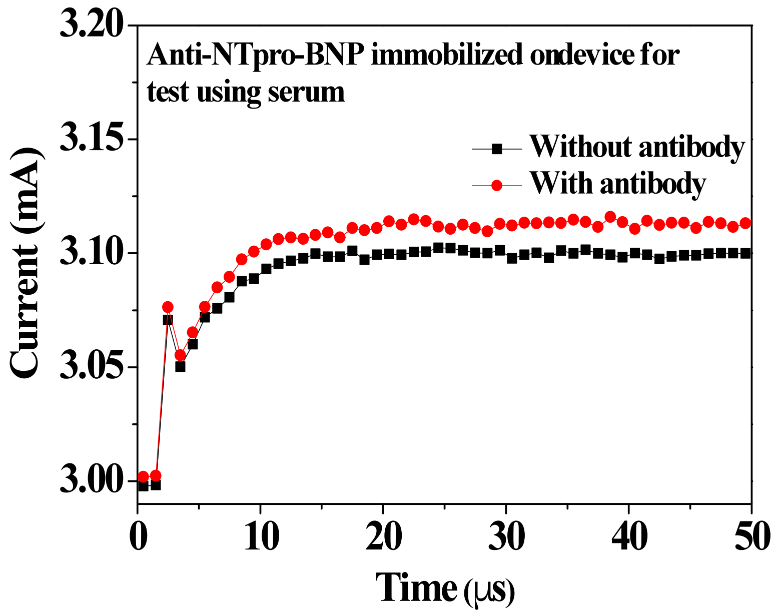

Figure S5 Successful immobilization of antibody to NT-proBNP on the device used for testing with clinical human serum samples.

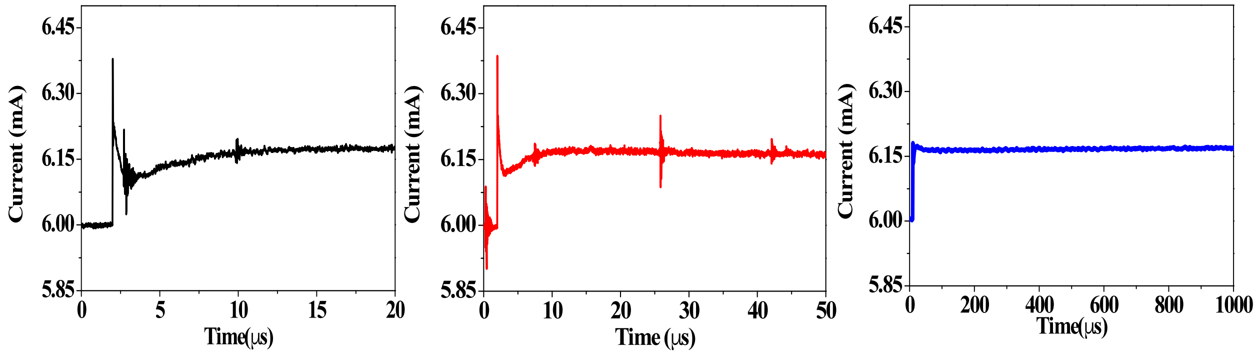

Figure S6 Investigation of different applied gate pulse bias times: 20 μs, 50 μs and 1ms. All of them reach steady state at about 10 μs and remain steady for the length of the applied pulse.

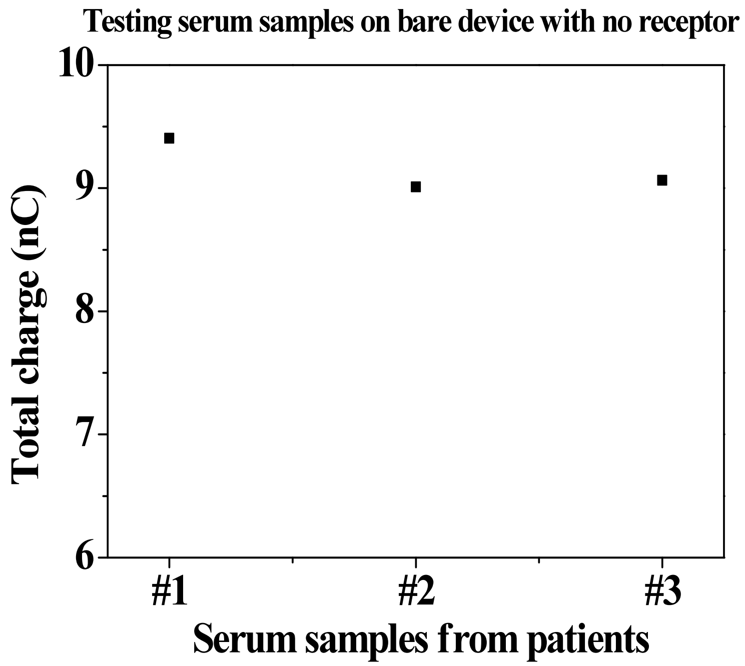

Figure S7 Background electrical response for sera from different patients. Drain current for each sample is measured on a bare device with no receptor immobilized. Total charge for three different samples from different patients indicates that the current response to the background protein matrix in serum is the same for different serum samples.
